# Centering the Inner Experience of Autism: Development of the Self-Assessment of Autistic Traits

**DOI:** 10.1089/aut.2021.0099

**Published:** 2023-03-13

**Authors:** Allison B. Ratto, Julia Bascom, Sharon daVanport, John F. Strang, Laura G. Anthony, Alyssa Verbalis, Cara Pugliese, Nicole Nadwodny, Lydia X.Z. Brown, Mallory Cruz, Becca Lory Hector, Steven K. Kapp, Morénike Giwa Onaiwu, Dora M. Raymaker, John Elder Robison, Catriona Stewart, Ren Stone, Emma Whetsell, Kevin Pelphrey, Lauren Kenworthy

**Affiliations:** ^1^ Center for Autism Spectrum Disorders, Division of Neuropsychology, Children's National Hospital, Washington, DC, USA.; ^2^ Autistic Self Advocacy Network, Washington, DC, USA.; ^3^ Autistic Women & Nonbinary Network, Lincoln, NE, USA.; ^4^ Gender and Autism Program, Children's National Hospital, Washington, DC, USA.; ^5^ Department of Psychiatry, University of Colorado School of Medicine, Anschutz Medical Campus, Denver, CO, USA.; ^6^ Department of Developmental Medicine, Boston Children's Hospital, Brookline, MA, USA.; ^7^ Department of Disability Studies and Women's and Gender Studies, Georgetown University, Washington, DC, USA.; ^8^ Private Consultant, Hitachiomiya, Ibaraki, Japan.; ^9^ DEIB and Disability Consultant, Evolving Skye, LLC, Levant, ME, USA.; ^10^ Department of Psychology, University of Portsmouth, Portsmouth, UK.; ^11^ Center for the Study of Women, Gender, and Sexuality, Rice University, & Autistic Women & Nonbinary Network, Houston, TX, USA.; ^12^ School of Social Work, Portland State University, Portland, OR, USA.; ^13^ Neurodiversity Working Group, The College of William and Mary, Williamsburg, VA, USA.; ^14^ Private Consultant, Ashfield, Dunblane, Scotland.; ^15^ Autistic Women & Nonbinary Network, Washington, DC, USA.; ^16^ Private Consultant, Portland, OR, USA.; ^17^ Department of Neurology, University of Virginia, Charlottesville, VA, USA.

**Keywords:** autistic traits, autistic phenotype, community-based participatory research, CBPR, self-report, autism, adults, qualitative research

## Abstract

Current tools for identifying autism are critiqued for their lack of specificity and sensitivity, especially in autistic people who are older, have higher verbal ability or significant compensatory skills, and are not cisgender boys. This may reflect the following: the historical focus of autism research on White (cisgender) male, upper and middle class children; limited interest in the inner, lived experience of autism; and the predominance of a deficit-based model of autism. We report here on the first attempt of which we are aware to develop a clinical self-report measure of autistic traits *as described by autistic people*. We believe this is an advance in methodology because prior work in the development of autistic trait/diagnostic measures has prioritized the perspectives of nonautistic clinicians and scientists. The measure was developed under the leadership of two autistic researchers and constructed by leveraging descriptions of autism by autistic people to generate items designed to encompass the range of the autistic experience, using strength-based, accessible language. The team utilized iterative feedback from a panel of autistic experts to refine and enhance the measure, called the Self Assessment of Autistic Traits (SAAT). It is intended for people 16 years or older and uses a format that is designed to increase its accessibility and acceptability for autistic respondents. Future work will report on the preliminary psychometrics of the SAAT, with a long-term goal of advancing our understanding of the inner autistic experience and enhancing the clinical and scientific assessment of autism.

## Community brief

**Why is this topic important?:**

Some people, especially older people, and those who can “mask” their autism, are missed by the current autism assessment tools. This can keep them from getting supports or getting connected to autistic communities. This can harm their well-being and independence. The tools we currently have to assess autism are important, but they were not developed with people who represent the full range of genders, ages, abilities, and cultural identities that characterize autism. Furthermore, current tools emphasize behaviors that other people observe, for example, making eye contact, and do not fully explore the lived or inner experience of autism.

**What is the purpose of this article?:**

This article describes the first attempt we know of to begin developing a self-report measure of autistic traits *as described by autistic people*.

**What did the authors do?:**

The authors started by reading what autistic people had to say about autism. They used those readings to come up with initial ideas about autistic experience. Then they used those ideas to write questions for a questionnaire about autistic traits. They asked autistic experts to review the questionnaire and made changes based on what they said.

**How did the authors work together?:**

This project was led by two autistic researchers who worked with a team of nonautistic researchers experienced in different research methods. A panel of autistic experts, including both autistic scientist and community leaders, also provided important input. Some of those methods were community-based research, Delphi panels, cognitive interviewing, and measure development. The research team made decisions together. The autistic researchers made the final decisions if there was disagreement.

**What did they produce?:**

They produced a preliminary version of the Self Assessment of Autistic Traits (SAAT). The SAAT is a questionnaire that asks if a person has common autistic experiences and traits. It has 58 items that are written with the aim of being respectful and using accessible language. The questionnaire is designed to work with common autistic thinking styles.

**How will this help autistic adults now or in the future?:**

The long-term goal is to create a reliable and valid self-report questionnaire that people 16 years old and older can complete to measure their autistic traits. We believe that this could be an important tool for advancing our understanding of the inner autistic experience of autism. This could improve how we assess autistic adults and how we research and think about autism.

## Introduction

Current tools considered gold standard for identifying autism are critiqued for their lack of specificity and sensitivity, especially in autistic people who are older, have stronger verbal ability or significant compensatory skills,^1^ and are not White cisgender boys.^2^ As a result, some autistic people go unidentified, or are misdiagnosed with other conditions,^3^ and do not get access to the treatments, supports, and connection with the autistic community that promote well-being and independence.^4^

There are many potential reasons for underidentification of autism, including the following: (1) a deficit-based model of autism that posits the nonautistic experience as the default and neglects the strengths of the autistic person, including how some strengths may be leveraged to compensate for areas of impairment^5,6^; (2) an overrepresentation of White, (assumed cisgender) male, and upper and middle class children across autism research,^7^ clinical, and diagnostic^8^ investigations; and (3) limited availability of self-report^9^ diagnostic tools that capture the inner experience of autism as autistic people describe it and in ways accessible for people with common autistic thinking styles.

Traditional autism research has overwhelmingly utilized a deficit-based model of autism, viewing autism solely through its impairments; however, autistic people also report that they experience autism as a source of deep meaning and joy,^6^ and that their abilities are highly impacted by their environment and the perceptions of others. The predominance of the medical model has limited consideration of autism within the social model of disability,^10^ in which disability is defined, or at least highly influenced by the social environment. As illustrated in the work of social scientists such as Grinker,^11,12^ societal perceptions of autism and autistic people have clear effects on the available supports and the autistic person's ability to find a place in society. Recent research indicates that autistic people more accurately read each other's emotions and have more successful social interactions with each other than do nonautistic people attempting to understand or interact with autistic people.^13,14^

Autistic people have also been shown to have important cognitive strengths compared with nonautistic people, for example, in visual processing, attention to detail, deep focus, and musical perception.^6,15^ Autistic people themselves have also described common strengths in memory,^16,17^ creativity,^6,18^ honesty,^6^ and empathy.^6^ These strengths are important signifiers of the autistic experience that are often de-emphasized in, or omitted from, traditional deficit-focused autism measures.

Research on autistic people has also long been plagued by a lack of representative diversity, both in demographic characteristics and in clinical presentation. Despite decades of public awareness campaigns and standardized screenings at well-child visits, non-White^19–21^ children and those of lower socioeconomic status^20,21^ remain underrepresented in autism diagnostic rates and continue to experience significant delays in diagnostic timing.^22–24^ Children who perform higher on standardized intellectual ability tests, and without obvious early developmental delays, also continue to be diagnosed later than their peers.^22^ In addition, evidence from epidemiological studies indicates that cisgender girls are diagnosed later than cisgender boys^25–27^ and experience greater delays in the time from an initial evaluation to receiving an autism diagnosis.^28^ There is also mounting evidence of elevated rates of co-occurring gender diversity and autism,^29,30^ which may impact the manifestation of autism and thus delay diagnosis.^31^

These factors have contributed to something of a tautology intrinsic to the field of autism research. Namely, we have developed our diagnostic tools and understanding of autism in highly homogenous samples,^32^ and then excluded from research any individuals who do not meet the criteria on diagnostic tools developed in these limited samples. Thus, while much of the rigorous research conducted to date finds minimal differences in the manifestation of autism based on factors such as sex^33^ or ethnoracial identity,^34^ these findings must be interpreted in light of the limitations of relying on research tools not developed within these populations.

An autism diagnosis is made by gathering evidence of *observable impairments* through developmental history and direct, behavioral evaluation with the individual.^35^ This contrasts with other psychological conditions (e.g., depression, anxiety), which also utilize, and often prioritize, self-report of the individual's inner experience, particularly for adolescents and adults. The validity of self-report in autism has often been questioned, based on the assumption that autistic people cannot accurately report on their inner experiences due to deficits in social–emotional insight,^36^ but these concerns are not empirically supported in many instances.^9,37,38^ Although self-report is not highlighted as critical to the diagnostic process, there are published self-report measures of autism available and in use.

One commonly used tool is the Social Responsiveness Scale-2: Adult (SRS-A),^39^ an adapted version of the SRS, which was originally designed as a parent-report measure. Similar to the parent-report version it is based on, the SRS-A tends to focus primarily on observable autistic traits and often asks the respondent to compare themselves with (presumably nonautistic) others. The Autism Quotient^40^ and the Ritvo Autism Asperger Diagnostic Scale-Revised^41^ were both developed as self-report measures of autism in adults, and thus avoid some of these flaws, but they were developed without autistic input. There are two more recently published measures that show important promise. The Autism Clinical Interview for Adults^42^ is an extensive interview with a companion questionnaire, developed with input from clinical researchers, diagnostic clinicians, and one autistic adult.

Similarly, the Comprehensive Autism Trait Inventory,^43^ which is a self-report questionnaire, was also developed with input from autism researchers, autism clinicians, and a focus of autistic college students. It is also notable that many different autistic people have developed descriptions and checklists of the autistic experience, which have been shared online but have not yet been published as standardized tools to be used in research and clinical practice. Without assessment of the inner experience as described by autistic people, clinicians may miss more subtle, covert, or internalized signs of autism. It is autistic people themselves who will be best able to inform measurement of that inner experience. For example, autistic people have written about the complex ways in which they navigate the social world to compensate for their challenges.^44,45^

Researchers have recently begun attending to these personal experiences, leading to a rising interest in the phenomenon of “camouflaging” (also referred to as “masking”),^44^ which has been long discussed among autistic people. Camouflaging is an emerging and somewhat controversial concept^46–48^ in autism research, which broadly refers to compensatory behaviors (whether conscious or unconscious) an autistic individual uses to navigate social expectations based on neurotypical standards.^49^ The behavioral and cognitive differences associated with camouflaging may also contribute to an autism diagnosis being missed or delayed. Camouflaging has been mostly strongly associated with possible female (or gender-diverse) phenotypes of autism and as a potential contributor to sex-based disparities in diagnostic rates,^1,50^ but studies on this process have shown that it occurs across genders.^51,52^ Notably, the most prominent tool for assessing camouflaging is the Camouflaging Autistic Traits Questionnaire,^53^ a self-report questionnaire, developed on the basis of qualitative interviews with autistic people about this phenomenon.

Autism research has been limited, then, by deficit-based conceptualizations, developed on restricted samples, and with limited input from autistic people themselves. This article describes an effort to address these problems by using the following conceptual framework to develop a new self-report questionnaire of autistic traits, which could eventually be used to improve clinical autism screening in adults (particularly those who may camouflage and thus be missed by traditional tools), to allow individuals to self-screen as part of a process of personal insight and self-empowerment, and to enable researchers to better understand the inner experience of autism to inform future research, by augmenting the information captured by more traditional diagnostic methods. This work was built upon the following three key principles.

### Engage autistic people with diverse demographic backgrounds and autistic identities and emphasize inclusive rather than reductive methods to represent the heterogeneity of autism

The research team emphasized purposeful inclusion in the demographics and lived experience of the autistic experts we engaged in this project—both through an initial internet search of influential autistic writings and in the recruitment of the panel of autistic experts who reviewed and augmented the research team's work. The research team did not rely solely on consensus to make decisions. Instead, the team used a multiple perspectives approach^54^ to allow the broadest extrapolation of items, thus honoring the complexity and heterogeneity of autism. Throughout the research process, new perspectives that did not fit with the preexisting or majority views were considered potentially meaningful expansions of the concepts under consideration, rather than as “nuisance” data to be excluded in favor of parsimony.

### Prioritize autistic perspectives

Information from autistic people about their own experience of autism represents a largely untapped resource for enhancing clinical and research tools for diagnosis and phenotyping and may “have far-reaching and disruptive effects on basic autism science research.”^55^ Autistic adults are sometimes asked to complete self-report versions of common questionnaires about their experiences and traits. To fully capture the inner experience of autism, however, autistic people need to define the questions asked,^5^ as well as the answers given. Our goal was to center autistic perspectives to generate a measure emphasizing the lived experience of autism, rather than the ways in which autistic people are observed to differ from nonautistic people. Existing self-report measures often require autistic people to rate themselves in relation to “others” (i.e., neurotypical people) or to describe how “others” see them, centering the neurotypical experience of autism, rather than the autistic experience.

This may be a particularly difficult demand for late-diagnosed individuals, who spend much of their lives unaware that they are not neurotypical and thus that their experiences may differ from those of neurotypical people.^45^ To ensure that the autistic experience was appropriately centered, we brought together a collaborative team of autistic researchers and nonautistic clinical researchers, in which the autistic researchers were considered the authentic experts and leaders of the process. Internet-disseminated writings by a diverse range of autistic people about the inner autistic experience provided the initial source material for our work, and a team of autistic experts reviewed and augmented the work of the research team, to ensure that autistic voices were prioritized throughout every stage of the research process.

The team drew from existing models of community-based participatory research (CBPR) when developing communication strategies within the research team, particularly the work of Nicolaidis, Raymaker, and other members of the Academic-Autistic Spectrum Partnership in Research and Education (AASPIRE) partnership,^56,57^ who have advanced the methods for CBPR with autistic self-advocates. Ground rules were set for the conduct of meetings and communication methods following review and discussion of the AASPIRE process.^56,57^ Our process also differed from CBPR, as the team was led by autistic researchers collaborating with “traditional” autism clinical researchers, rather than a partnership of lay community members and traditional researchers.

### Highlight autistic strengths and the impact of societal structures on autistic people

Happé and Frith^58^ note that autistic-led research naturally leads to a change in how we understand autism, shifting “from conceptualising autism purely as a ‘developmental disorder,’ to recognising a neurodiversity perspective.” The philosophy of neurodiversity was important to our thinking, meaning that we recognized that neurotypical thinking should not be considered the default from which autistic thinking should be judged.^6^ Thus, in developing this new measure, we used a framework of “autistic experience” rather than “autism diagnosis.” We sought to create a measure that would identify autistic people based on the strengths, unique experiences, and challenges commonly associated with autism, as well as describe its divergences from an assumed societal norm. We also sought to inform our understanding of autism through the framework of the social model of disability to recognize that many challenges commonly associated with autism result from a lack of appropriate societal supports for autistic people.^12^

Our goal was to create a measure in which autistic people could describe themselves through their own modalities of thinking and interacting with the world, including ways in which autistic people succeed and find joy in a world that is often poorly adapted to their needs.

## Methods and Results

**FIG. 1.**
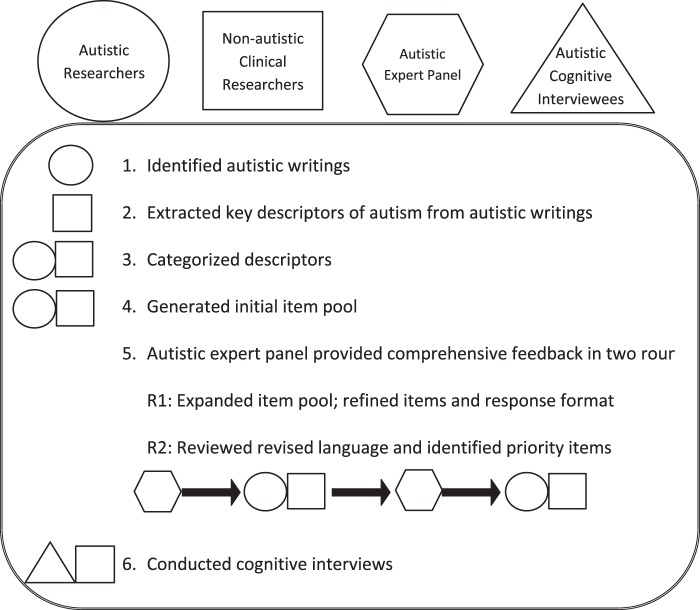
Process and roles in the development of the Self-Assessment of Autistic Traits Item Pool.

Following Institutional Review Board-approved protocol Pro00010580, the autistic-led team of researchers collaborated to develop a self-report questionnaire of autistic traits and experiences for people 16 years old through adulthood called the Self Assessment of Autistic Traits (SAAT). We constructed the measure by leveraging written, publicly available descriptions of autism by a diverse group of autistic people to generate items designed to encompass the diversity of the autistic experience, using strengths-based, accessible language. The team utilized feedback from autistic experts throughout the process to refine and enhance the measure (Fig. 1). Notably, this project was originally conceptualized as one led by “traditional,” nonautistic clinical researchers, with collaboration from nontraditional, nonacademically affiliated autistic researchers.

Following in the footsteps of groups such as the AASPIRE partnership, the dynamics shifted as the work began, such that the autistic researchers rapidly became fully included partners and ultimately leaders of the research team. The protocol engaged the following four groups of individuals, as described here.

### The core research team

Two autistic researchers (i.e., who themselves are autistic; hereafter referred to as “Autistic Lead Researchers”) led the team, which also included six nonautistic clinical researchers. Autistic Lead Researchers: The two Autistic Lead Researchers (J.B. and S.d.V.) are each directors of autistic advocacy groups in the United States and have extensive experience partnering with traditional academic researchers, including for the development of assessment tools. The Autistic Lead Researchers have expertise in how the autistic community in the United States talks about autism, intersectionality in disabled people, and the use of surveys and written communication with autistic people.

Nonautistic Clinical Researchers: The six Nonautistic Clinical Researchers are all employed in academic medical centers and have conducted traditional autism research and clinical work with autistic youth and adults. They represent expertise in autism diagnosis (L.G.A., A.R., C.P., and J.F.S.), measure development and evaluation (L.K., J.F.S., A.R., and L.G.A.), autism presentation in cisgender females (L.K., C.P., A.R., A.V., and L.G.A.) and gender-diverse people (J.F.S. and L.G.A.), CBPR techniques (J.F.S., L.G.A., L.K., A.R., C.P., and A.V.), and use of the Delphi methodology (J.F.S., L.G.A., and L.K.). See our Open Science Framework page for a description of how the team collaborated (https://osf.io/5krxy/?view_only=8f5bbbc964aa44ae983694923468ff8f).

Core Research Team Identities and Positionality: The core research team included some diversity in sociodemographic variables but was primarily composed of White cisgender women (Table 1). All were located in the United States, with most in the Washington, DC metropolitan area, as well as one member in Colorado and one in Nebraska. The work began formally in 2017 and continues to the present.

**Table 1.** Self-Reported Demographics of Autistic Expert Panel and Core Research Team

	Autistic expert panel (*N* = 19)	Core research team (*N* = 8)
Age in years^a^		
Mean, SD	35.58, 10.50	46.00, 10.71
Range	25–62	30–60
Race/ethnicity		
Asian/East Asian	2	0
Black	4	0
Biracial/multiracial	2	1
White	10	7
Unspecified	1	0
Gender^b^		
Agender/female	1	0
Cis man	1	0
Complicated	1	0
Female	6	6
Female/gender fluid	1	0
Male	5	0
Nonbinary	0	1
Nonbinary, genderqueer, agender	1	0
Trans male/demiguy	1	0
Trans nonbinary	1	0
Cisgender female or AFAB *(though my gender identity has been evolving)*	1	0
Questioning	0	1
Sexual orientation		
Bi/pan	1	0
Bisexual	4	1
Demisexual	1	0
Bisexual/demisexual	0	1
Gay	0	1
Heterosexual	7	5
Lesbian	0	1
Queer	3	0
Queer asexual	1	0
Queer omnisexual	1	0
Other	1	0

Nineteen of the 21 panelists provided demographic data.

^a^ Seventeen panelists provided numerical values for age.

^b^ Autistic expert panelists were not asked to provide designated sex at birth. For this reason, gender diversity status may not be represented in all of the responses. For example, if a panelist described their gender as “male,” there is no specification as to whether they are a cisgender or transgender man.

AFAB, assigned female at birth; SD, standard deviation.

**Table 2.** Changes Made to Self Assessment of Autistic Traits Item Pool Based on Expert Panel Review

	Expert panel Round 1	Expert panel Round 2	Autistic adolescent comprehension checks
Items submitted by research team for review	52	84	58
Changes following review			
Items edited	45	9	16
Items added	37	0	0
Items removed	5	26	0
Unchanged items	2	49	42

*Note:* In outreach to the Delphi experts about this article, following completion and initial piloting of the item pool, the research team discovered that one of the Delphi experts was not autistic and had participated despite instructions that their participation was contingent upon being autistic. The team also discovered that in Round 2, the team had reviewed only the qualitative, and not the quantitative, feedback from another one of the Delphi experts. The team recalculated item endorsements with the nonautistic panelist removed and the omitted panelist added and determined that these errors did not impact decisions to remove or maintain any items as presented in Table 2, nor did feedback from the nonautistic panelist impact the revision or wording of any items. Data presented here were unaffected by the changes to the panel.

### Autistic expert panel

The autistic lead investigators purposively invited a panel of international autistic experts to represent autistic people who (a) are cisgender and gender diverse; (b) communicate through multiple methods; (c) are diverse in ethnoracial identity, geographic location, and national origin, and (d) include a broad age range (Table 1). All had expertise in autistic experiences beyond their own (e.g., through roles in research or advocacy). Of 24 invited experts, 20 agreed to participate and completed the first-round review of the SAAT item pool; 19 completed the second-round review. The autistic experts communicated with the research team through anonymous online surveys.

In addition, the team compensated the autistic experts for their time. The panel of autistic experts made significant contributions to the development of the SAAT and were invited to be authors on this article; some elected to participate in the construction of this article and are listed as authors and others elected to be acknowledged for their contributions instead.

### Cognitive interviewees

Five autistic adolescents (3 females, age range 15–19 years; 3 White/non-Latinx, 1 Black, 1 White/Latinx) without intellectual disability were invited by the Nonautistic Clinical Researchers to complete cognitive interviewing with the SAAT items in person or over the telephone with a member of the research team (L.K.) or a trained research assistant (N.N.). The team compensated the youth for their time with gift cards.

## Procedures

### Overview

The Autistic Lead Researchers gathered writings by autistic people and distilled them into themes. From these, the Core Research Team (Autistic Lead and Nonautistic Researchers) developed the items with extensive augmentation and editing from the Autistic Expert Panel. The Core Research Team and the Autistic Expert Panel iteratively exchanged ideas about constructs to include or exclude (Fig. 1).

### Step 1

The two Autistic Lead Researchers compiled a list of autistic-authored online writings (e.g., Blog posts, articles; see Supplementary Material S1 for a complete list of documents) that have been historically centered in the autistic community (i.e., widely read, shared, and referenced by autistic people). Although we were unable to directly confirm with each author of the internet writings, it appears (based on publicly available information in the writings or surrounding texts) that the internet authors were of diverse genders, support needs, and communication modalities, but they were predominantly White (see Supplementary Material S1 for a complete listing of writings).

### Step 2

The Nonautistic Clinical Researchers each read overlapping subsets of the online writings compiled in step one and independently extracted key themes regarding the inner experience of autism, which were then shared in extended, in-person meetings of the Core Research Team. From the themes, the team worked to develop descriptors and then items, with an added focus on including descriptors not currently emphasized in traditional autism conceptualizations.

### Step 3

To ensure adequate coverage of a broad range of autistic experiences, the Core Research Team organized descriptors into categories, using the Autistic Self Advocacy Network (ASAN) “About Autism” framework (2018) as an initial guide (Supplementary Material S2). The team used a multiple perspectives approach^54^ to allow the broadest extrapolation of items, thus honoring the complexity and heterogeneity of autism. Descriptors were either associated with one of seven components identified in the ASAN framework or determined to represent a new category. Decision-making regarding the classification of the descriptors relied on the process described on our Open Science Framework page (https://osf.io/5krxy/?view_only=8f5bbbc964aa44ae983694923468ff8f), with the final decision-making power resting with the Autistic Lead Researchers. In total, 13 categories (one of which included 5 subcategories) were identified and consolidated into four overarching domains (Table 3: Domains 1–4).

**Table 3.** Domains (with Categories and Subcategories) Developed by Research Team and Expanded Through Modified Delphi for Item Development

1. Sensory/motor loop—
a. Different sensory experiences
b. Motor skills
c. Repetitive movements
2. Strength in specificity
a. Deeply focused thinking and passionate interests in specific subjects
b. Need for consistency, routine, and order
3. Social communication
a. Difficulties in using language
b. Difficulties in using nonverbal communication
c. Social syncing and compensation
d. Different social needs
4. Executive function and related traits
a. Regulation and modulation
i. Cognitive regulation/flexibility
ii. Strong empathy
iii. Overload
iv. Burnout
v. Variability of abilities
b. Autistic inertia
c. Uneven skills
d. Daily living skills
5. Negative community context (5th domain added following expert panel review, Round 1)
a. Systemic discrimination
b. Chronic social trauma

### Step 4

The Core Research Team then converted the descriptors into items, creating an initial pool of 52 items, to be rated based on the degree to which the respondent felt the item represented the autistic experience (Table 4). Item wording was guided by recent work^59^ on the creation of surveys that are accessible to autistic people, and the prior experience of the two Autistic Lead Researchers. The team chose a slider scale format for responding to items, in which respondents moved a slider along a scale to indicate the degree to which the item was “Not True,” “In the Middle,” or “Completely True” of them, based on their current experiences.

**Table 4.** Item Endorsement at Second Round of Delphi Expert Review (Abbreviated Item Content)

	Original (incorrect) endorsement	Updated (correct) endorsement
*Items retained in final SAAT item pool (endorsed >70%)*		
My senses are extreme.	100	100
I like to do certain things over and over again.	100	100
I don't know how to have the friendships I want.	95	89
When I feel overwhelmed, I might have a meltdown, or I might need to run away.	95	95
When I feel overwhelmed, I might shut down.	95	95
I have intense, passionate interests.	89	89
I know how to take care of myself, but I might forget to do things.	89	89
I get overwhelmed by things my body senses.	84	79
I have strong urges to make certain movements, even if I don't want to.	84	84
I often notice details that other people don't.	84	84
I prefer to follow a routine.	84	89
I can't always understand what people say when they talk to me.	84	89
When I'm thinking a lot about something, it's hard for me to switch to a new topic.	84	84
Transitions are hard.	84	84
I can get so focused on something that I stop being aware of the rest of the world.	84	84
I get overwhelmed by things that seem easy for other people.	84	84
It is hard for me to do all the things I need to do in the day to take care of myself.	84	84
I am direct when I can communicate with people.	84	79
I am a literal thinker.	84	79
I process things much better one sense at a time.	79	84
I do my best when I know what to expect.	79	74
In a conversation, it is hard to understand other people and communicate what I mean.	79	74
I need to rest and recover after something really overwhelming.	79	79
I can spend hours trying to start doing something that I want to do, but I can't get started.	79	79
Just because I can do one thing doesn't mean I can do similar things.	79	84
It's hard for me to notice what my body needs.	74	74
It is hard for me to come up with my own words.	74	68
It is easier for me to have conversations online or via text.	74	68
I can count on other people to understand my tone of voice, body language, and face.^*^	74	74
I usually don't know how I feel until I'm overwhelmed or time has passed.	74	79
Some of my abilities vary from day to day.	74	74
In order to show what I am capable of, I need to do things a certain way.	74	68
I am good at recognizing patterns.	74	74
When something interests me, I can focus on it so much that I become an expert.	74	74
I am treated unfairly by others.	74	68
People get mad at me when I don't act the way they expect me to.	74	74
I am always on guard in case social situations go wrong.	74	74
When other people suffer, I feel it strongly.	74	74
I care a lot about other people being treated fairly.	74	74
*Items retained in final SAAT item pool (endorsed <70%)*		
Using language is automatic for me.^*^	68	68
It is easy for me to know what people mean by their tone of voice, body language, and face.^*^	68	68
Daily living tasks are harder for me than reasoning or logic tasks.	68	68
I love some sensations so much that I can get lost in them.	68	68
It is easy for me to know what other people think is important in a conversation.^*^	63	58
There are times I suddenly lose skills that I used to know very well.	63	68
I can't always do what I need to do on my own. I need another person to help me.	63	68
I figure out social situations by using logic, flowcharts, or other systems.	58	53
It's easy for me to break down a task or a goal into several steps.^*^	58	53
People tell me my experiences aren't real.	58	58
I have a hard time getting other people's attention when I'm in a group.	58	63
I say things I don't mean because I get stuck saying the wrong words.	58	63
Doing small tasks with my hands is always easy.^*^	53	53
I often practice what I want to say in conversations.	53	53
I make up my own ways for figuring out what people mean by their tone of voice, body language, age face.	53	58
It is easy for me to sync socially with groups of people.^*^	53	53
Doing big movements with my body is always easy.^*^	47	53
I can't always do what I need to do, because it feels like my mind and my body are disconnected.	47	47
Learning new ways to move my body or my hands is always easy.^*^	37	42
*Eliminated items*		
I need more help to do something if it happens outside of my routine.	68	68
I have unusual talents.	68	68
I get overwhelmed by people.	68	63
It's hard for me to put my feelings into words.	63	58
I need to live with someone else, or have someone come and help me do things every day.	58	63
I am an independent thinker.	58	58
I don't care about social hierarchy.	58	53
Other people treat me like I am not a real person.	53	58
If I get too overwhelmed, I will have a meltdown or shutdown.	53	58
It is easier for me to use things like body language, tone of voice, or eye contact separately.	53	58
At some point in my life, falling or staying asleep has been a major problem.	53	47
I give my best answer when I know I have as much time as I need to respond.	53	47
I prefer to make friends online.	53	47
I like talking about facts and ideas more than social topics.	47	47
I learn what to do in social situations by watching other people. Then, when I'm on my own, I practice.	47	53
I learn new ideas best when I start with specific details. Then, I work up to the big picture.	47	47
I can think faster than I can talk.	42	47
My mind and body have a hard time switching from asleep to awake.	42	42
I have been told to ignore my own needs to make other people happy.	37	42
I really like to have fun with words and phrases.	37	37
I copy things like movements, body language, or accents from other people.	32	37
I see things one part at a time.	21	16

^*^ Denotes reverse-coded items.

### Step 5

After creating the initial item pool and survey instructions, the Core Research Team engaged the Autistic Expert Panel in an iterative, two-round exchange in which the panel used online surveys to provide feedback on the items, including how to improve items, what items were missing, and items to be excluded. Our approach used traditional Delphi procedures for anonymous surveys in which experts in a field provide qualitative and quantitative feedback. The Core Research Team intentionally made two major modifications to the standard Delphi process^60^: (1) the Core Research Team analyzed and processed the expert feedback to make changes to the itemset themselves, to which the Autistic Expert Panel then reacted and (2) the Core Research Team made final decisions that at times were not based on panel consensus.

We felt that these modifications were necessary to retain items that might be important to a smaller subset of autistic people (e.g., Black, Indigenous, and People of Color [BIPoC] people, nonspeaking autistic people), and consistent with our principle of prioritizing the autistic perspective, given that the Core Research Team was led by the Autistic Lead Researchers, who held the final decision-making power.

#### Round 1

In the first round, the Core Research Team asked the Autistic Expert Panel to respond to a single yes/no question (Does QUESTION X capture an important aspect of being autistic?) and invited them to provide additional, open-ended feedback if they wished for each item. The Autistic Expert Panel also provided input on the use of a slider scale (which 19 out of 21 experts approved), as well as open-ended feedback on important aspects of the autistic experience and on the survey as a whole. Key areas of feedback from the Autistic Expert Panel included ensuring clarity in item wording, emphasizing positive framing of traits, and avoiding the positioning of neurotypical experiences as the standard or norm. In addition, they provided important suggestions for new descriptors, highlighting autistic joy, independence from social norms, strong empathy and altruism, the importance of intersectionality, experiences of being misunderstood by others, and impactful experiences with stigma and discrimination.

In response, the Core Research Team revised 45 items (Table 2) to make items accessible and appropriate to a sixth grade reading level, to include concrete examples, and to frame items from an autistic perspective, using positive wording. For example, an item phrased: “I have urges to hurt myself or make certain movements, like to hit my head or touch lamp posts. I don't want to, but they are very hard to control” was rewritten as “I have strong urges to make certain movements, even if I don't want to,” with several concrete examples provided below the initial sentence to improve clarity.

An item phrased “What other people think is important about a conversation or interaction is not always clear to me. I often don't know what the right thing to say is” was rewritten as “It is easy for me to know what other people think is important in a conversation” to improve positivity and center autistic perspectives. In addition, the Core Research Team added a fifth domain, *Negative Community Context*, to the original four (Table 3), in recognition of the pervasive emphasis on the impacts of stigma, bias, and discrimination on autistic people. At the end of Round 1, the research team provided a revised pool of 84 items to the autistic expert panel for review in the second round.

#### Round 2

Of the original 20 autistic experts, 19 participated in the second round. The experts responded to four Yes/No questions in regard to each item: (1) Does this match your personal experience of autism? (2) Do you think this matches the experience of many other autistic people? (3) Is the wording accessible? (4) Is this item essential to capture a core part of autism? and could also provide optional open-ended feedback. Questions 1 and 2 (above) were designed to address the fact that in the first round some experts were unsure whether to respond based on their *personal lived experience of autism* versus their *expert knowledge of the autistic experience broadly.*

The Core Research Team measured the proportion of the expert panel that endorsed each item as essential (see Table 4); items with <70% endorsement by the experts were recommended to be dropped. Consistent with a multiple perspectives approach, the Core Research Team made a few exceptions to retain items considered crucial for a subset of autistic individuals with less commonly observed traits or who were potentially marginalized with the community (e.g., autistic BIPoC; nonspeaking autistic people). This step was taken to account for the inequitable representation of these groups on the panel, and we commit to more equitable representation in the future to ensure that our research is high quality, meaningful, and generalizable. This resulted in an item pool of 58 iteratively revised descriptors of autism (see Table 2 for a summary of changes).

### Step 6

To ensure that the measure would be accessible to nonexpert users and youth, the Core Research Team invited five autistic adolescents to participate in cognitive interviews regarding the item pool. An interviewer asked the adolescents to read each item and then tell the interviewer what they understood the item to mean in their own words and how they would respond to the item themselves. The Core Research Team reviewed the transcriptions of the interview and made minor edits to 16 items to improve comprehension (Table 2).

## Outcomes

Based on the work in Steps 1–3 (review and discussion of autistic-authored internet writings), the Core Research Team generated a framework for developing items to assess the inner autistic experience, based upon four overarching domains, as noted above: (1) Sensory/Motor Loop, (2) Strength in Specificity, (3) Social Communication, and (4) Executive Functioning and Related Traits (Table 3). The Sensory/Motor Loop domain addressed sensory and motor experiences (both positive and challenging), such as having extreme sensory experiences or processing senses one at a time. The Strength in Specificity Domain highlighted strengths in identification of patterns and details and developing deep knowledge. The Social Communication Domain focused on the unique ways in which autistic people communicate and utilize language (e.g., strategies for understanding nonverbal communication, communicating in direct or literal ways), as well as experiences within social interactions and relationships (e.g., developing desired friendships, understanding others' thoughts and feelings).

The Executive Functioning and Related Traits Domain was a diverse category, capturing the ways in which autistic people experience and navigate their world and personal lives. This included items tapping experiences of transitions, overload, and burnout, as well as how autistic people best attend to daily tasks (e.g., use of timers and routines) and show their skills (e.g., by writing versus speaking aloud). Several items within these domains included aspects of autism that are not directly captured with the current diagnostic or phenotyping tools, such as: “autistic inertia,”^61^ which refers to a difficulty linking intention and motor plan to initiate an activity; regression in abilities outside of early childhood; the way that automatic or scripted language can override communicative intent; and the impact of overload on the functioning of autistic people. The modified Delphi process validated the research team's four domains of autistic experiences.

As reflected in Table 2, the autistic experts deemed the vast majority of the items to be accurate and important descriptors of autistic experiences. They also provided feedback to expand content within each of the domains identified, indicating that these domains highlighted critical aspects of autistic lives. In addition to the four initial domains, the autistic expert panel identified a critical fifth domain that was missing from the initial framework, namely, that of Negative Community Context. This domain addressed experiences of systemic discrimination and chronic social trauma that autistic people often encounter,^62^ such as experiences of gaslighting or being the target of others' anger.

Furthermore, the Core Research Team used the intensive feedback provided by the Autistic Expert Panel to refine the content and wording of items for clarity and ensure that items within each domain and category reflected the range and complexity of the inner autistic experience, both positive and negative, from the autistic perspective. The final result was an innovative five-domain framework of the inner autistic experience, with a final item pool of 58 items to be piloted in the SAAT (Table 4).

## Discussion

We report here on the first attempt of which we are aware by a team of “traditional” (i.e., clinical, nonautistic) and autistic researchers to develop a self-report measure of autistic traits and the inner experience of autism *as described by autistic people*. We hope that it will eventually be used in adult autism screening in both clinical practice and research, as a means of augmenting traditional diagnostic methods. As the phenomenon of autism self-diagnosis^63^ becomes more common, the SAAT may also serve as tool to help potentially autistic adults gain personal insight and link to autistic community and other supports. Furthermore, we hope that this tool will be used in research to enable deeper investigations into the inner autistic experience so as to guide future research questions. Prior work in the development of autistic trait/diagnostic measures has largely prioritized the understanding and perspectives of nonautistic clinicians and scientists.

This model has thus relied on defining autism through the ways in which autistic people's behavior differs from “the norm” (i.e., neurotypical individuals), rather than the lived experiences of autistic people. In contrast, this project developed a measure using the inner autistic experience as the foundation, with continuous input from a diverse range of autistic people to guide both the content and response formatting. This required the combination and adaptation of several methods to enable the operation of a research team led by two autistic researchers working alongside nonautistic clinical researchers who had experience in measure development and autism diagnosis and phenotyping. The project also prioritized inclusion of the perspectives of autistic people with diverse gender and ethnoracial racial identities and modes of communication, as well as a variety of support needs, additional disabilities, and educational and employment experiences.

Many of these perspectives have been missing from previous investigations of autistic traits, and thus, these individuals may not be accurately classified by traditional autism diagnostic measures. Our methodology, driven from the ground-up and—throughout the process—by autistic experts, represents a profound departure from the methodology used for developing previously established tools, intending to enable new insights into the autistic experience.

The resultant item pool, the SAAT, adds components designed specifically to capture the inner experience of autism across the diverse population of autistic people. Thus, while there is some overlap in content between the SAAT and currently available self-report autism measures (e.g., preference for repetition, sensory differences), its centering in the lived experiences of autistic people led to the inclusion of autistic traits, which have been overlooked in previous phenotyping measures (e.g., autistic inertia, autistic burnout, temporary regression). These traits, if validated by others, may expand our ability to identify autism in a broad range of individuals whose experiences have been underrepresented in the development of the most commonly used diagnostic tools. The iterative process that centered the autistic perspective identified four initial domains, which, along with individual items, were validated through a modified Delphi process leveraging the expertise of autistic people.

This process then expanded that framework to five domains that further elucidate the ways in which the inner experiences of autistic people both align (e.g., preferring routines, difficulty with conversations and friendships) and differ (e.g., experiences of gaslighting, inconsistency in demonstrating skills, motor planning difficulties, autistic burnout) from traditional conceptualizations of autism. This may reveal new targets for research that can enhance our understanding of autistic lives and well-being.

Moreover, the SAAT moves beyond a deficit-based model to include autistic strengths and joy, such as in noticing details and patterns, strong empathy and honesty, and creative problem-solving. Although currently available self-report measures include some positive wording and framing of autistic strengths, this was a true priority in the development of the SAAT and was emphasized and improved through the input of the Autistic Expert Panel. In framing items about challenges, the SAAT emphasizes how autistic people are most successful (e.g., by practicing language in advance, finding unique ways to understand nonverbal communication, or demonstrating knowledge in specific ways), rather than the challenges themselves.

By more accurately capturing the inner experience of autism, the SAAT aligns well with a neurodiversity perspective because it presents some traits that are often pathologized (e.g., restricted interests, repetitive behaviors) in a neutral or positive manner and represents their value in the inner experience of autism. It also queries autism traits without making comparisons with an implicit neurotypical standard whenever possible and includes items that capture experiences of prejudice and misunderstanding.^62^ The inclusion of these experiences recognizes the role that communities can play in discriminating against autistic people, intentionally or unintentionally.^12^ This potentially makes the measure more acceptable to autistic people and enables the SAAT to more accurately capture the complexity of the autistic experience by framing autistic traits through an autistic lens (e.g., describing intense interests as deep passions, rather than all-consuming interests that eclipse all other interests).

Because autistic people both developed (as Autistic Lead Researchers) and reviewed (through the Autistic Expert Panel) the SAAT item pool, it uses a format that is consistent with common autistic thinking styles, which we believe will increase its accessibility. For example, many items include specific examples, in recognition of the many autistic people who benefit from detailed, concrete information when interpreting general, or abstract, language. It utilizes a slider scale to avoid asking respondents to select one of several possibly constraining or ambiguous categories (e.g., often, sometimes, or never). The reading level of most items is below sixth grade, which further increases accessibility across a broader range of reading and language ability levels than the prior self-report measures.

The Core Research Team used simple sentence constructions to support comprehension and also considered the impacts of pragmatic language difficulties, to ensure that the SAAT does not require readers to interpret implicit or figurative language to understand items. At times, this prohibited use of lower reading level language, but the intention of this approach was to make the measure more accessible overall to an autistic population. These measures are consistent with prior adaptations made in CBPR with autistic people.^56,59^

Reflecting on the process that produced the SAAT, the Core Research Team was humbled to realize that without the expert panel guidance we would have failed to live up to many of our stated principles for developing the SAAT. In its first iteration, the Autistic Expert Panel provided feedback that, despite the research team's intentions, some items portrayed autistic people negatively and caused respondents to feel badly about themselves, which, in addition to raising ethical concerns for the research team, had the potential to affect how respondents interpreted the subsequent items. Some items seemed to posit the neurotypical experience as the standard or asked autistic people to compare their experiences with that of a neurotypical person (e.g., asking them to rate their own level of difficulty with a task to that of “most people”). Other items were imprecise in their wording and/or unclear in their meaning to the autistic experts.

The Core Research Team was grateful for the Autistic Expert Panel's help in correcting those oversights and improving the measure. The panel also identified important aspects of the autistic experience (e.g., incidents of discrimination and bias by neurotypical people) that the team had not captured in the first iteration of the SAAT.

Although the SAAT and our development process include many important and innovative strengths, we acknowledge that there were relevant limitations as well. The team made efforts to include diverse autistic perspectives in our process, but our process failed to include some groups (i.e., most notably autistic people with intellectual disability) and underrepresented other groups (e.g., BIPoC autistic people). Furthermore, the authors of the internet writings from which the research team initially extracted the themes were predominantly White. Although the Autistic Expert Panel who augmented and revised the SAAT item pool was considerably more racially diverse, and helped somewhat to correct for this early imbalance, we nonetheless recognize that our own internal bias, influenced by the systemic racism that has historically erased BIPoC people in research,^7^ no doubt impacted our initial approach.

As we recognized these initial mistakes, we sought to make corrections in a way that would also ensure our work remained bound to the integrity of research principles. We further commit to centering future research that prioritizes and intentionally includes the BIPoC community at the onset of all work. Similarly, we recognize that the Autistic Expert Panel underrepresented both autistic people with high support needs and nonspeaking autistic people. These are important subsets of the autistic community that have been increasingly overlooked and excluded from autism research.^6^ As we move forward in our research and development of this tool, we commit to doing the necessary work to ensure that eventually this tool is inclusive of and accessible to autistic people with a range of support needs and communication modalities.

We also performed a relatively small number of cognitive interviews. Although the cognitive interviews did lead to important changes in the measure, additional cognitive interviewing may well have revealed other unclear items.^64^ This present work also focuses solely on the development process. Future work will report on the preliminary psychometrics of SAAT. Critical to psychometric work is the piloting of the SAAT with diverse nonexpert autistic people, who may respond to items about themselves differently than do autistic experts. As our team continues to develop and refine this measure, we are hopeful that it can significantly advance our understanding of the inner autistic experience and enhance our clinical and scientific assessment of autism.

Finally, most of our nonautistic clinical researchers (L.K., C.P., A.R., J.F.S., and A.V.), similar to many autism researchers, are trained primarily in childhood and adolescence and indeed work in academic medical centers for children and adolescents, although one (L.G.A.) is trained in life span approaches and works predominantly with adults. Moreover, several of the nonautistic clinical researchers (J.F.S., C.P., L.K., and L.G.A.) have a substantial focus on the transition to adulthood and young adult experiences as well. Important perspectives on adult autism were also garnered through the work of the autistic lead researchers (J.B. and S.d.V.) and the input of our expert autistic panel.

This highlights a broader challenge in the field that clinical and research training in autism is generally associated with pediatric settings, and there are limited opportunities for clinicians and researchers focused on adulthood to access autism training. We are hopeful that the SAAT can contribute to the ongoing movement to recognize that autistic children become autistic adults, from whom there is still much to be learned.

## Supplementary Material

Supplemental data

Supplemental data

Supplemental data

## Supplementary Material

Supplementary Material S2

[Supplementary-material SD3]
