# Supplementary material for: Centering the Inner Experience of Autism: Development of the Self-Assessment of Autistic Traits
Source: Autism Adulthood. 2023 Mar 13;5(1):93–105. doi: 10.1089/aut.2021.0099 (PMC10024271; doi:10.1089/aut.2021.0099)
Supplement: Supplemental data [file Suppl_MaterialS1.docx]

- - - 1. **Autistic-Authored Online Writings**

| Title | Author/Source* |  |
| --- | --- | --- |
| Welcome to the Autistic Community | Autistic Self Advocacy Network (https://autistic-advocacy.org) |  |
| About Autism | Autistic Self Advocacy Network  (https://autistic-advocacy.org) |  |
| What is Autism Factsheet | Autistic Self Advocacy Network (https://autistic-advocacy.org) |  |
| AWN Welcome Information for Parents | AWN (https://awnnetwork.org) |  |
| AWN Welcome Information for Autistic Women | AWN (https://awnnetwork.org) |  |
| What Every Autistic Girl Wishes Her Parents Knew | AWN (https://awnnetwork.org) |  |
| Cultural Commentary: Up in the Clouds and Down in the Valley: My Richness and Yours | Amanda Baggs (https://dsq-sds.org/article/view/1052/1238) |  |
| Inertia: From Theory to Praxis | Anna Sullivan (Inertia: From Theory to Praxis) |  |
| Inclusive autistic traits | Autisticality (https://autisticality.com/2016/03/21/inclusive-autistic-traits/) |  |
| Autistic-Created Alternative Autistic Criteria | d&d cryptid (https://politeyeti.tumblr.com/post/16448184988/autistic-created-alternative-autism-criteria) |  |
| Passive Plants | DJ Savarese (https://iowareview.org/from-the-issue/volume-47-issue-1-%E2%80%94-spring-2017/passive-plants) |  |
| Bridging the Gaps: An Inside-Out View of Autism: (Or, Do You Know What I Don't Know?) | Jim Sinclair (in *High-Functioning Individuals with Autism*, edited by Eric Schopler and Gary B. Mesibov. Plenum Press, New York, 1992). |  |
| Excuses to be a jerk. (for BADD) | Mel Baggs (https://ballastexistenz.wordpress.com) |  |
| On growing up with strange sensory reactions, and the difference between passing and being passed off. | Mel Baggs (https://ballastexistenz.wordpress.com) |  |
| Oak manifesto | Mel Baggs (https://withasmoothroundstone.tumblr.com/ |  |
| [Autistic Inertia: An Overview](http://unstrangemind.com/autistic-inertia-an-overview/) | [UNSTRANGEMIND](http://unstrangemind.com/author/unstrangemind/) (http://unstrangemind.com) |  |
| [Autistic Shutdown Alters Brain Function](http://unstrangemind.com/autistic-shutdown-alters-brain-function/) | [UNSTRANGEMIND](http://unstrangemind.com/author/unstrangemind/) (http://unstrangemind.com) |  |
| The ABCs of Stimming | Par la fenêtre (https://fuckyeahstimming.tumblr.com) |  |
| Atypical Autism Traits | and you did what you had to do (https://mostlyanything19.tumblr.com) |  |
| What is Autism in Plain Language | Autistic Academic (https://autisticacademic.com) |  |
| The Problem With The Way We Speak About Autism | Michelle Sutton (http://michellesuttonwrites.com) |  |
| The Problem With How We Treat Autism | Michelle Sutton (http://michellesuttonwrites.com) |  |
| The Problem with How We Diagnose Autism | Michelle Sutton (http://michellesuttonwrites.com) |  |
| Discourse and Semantics | Michelle Sutton (http://michellesuttonwrites.com) |  |
| Conceptualizing Autism | Anne Corwin (Document shared via Google Docs, which has since been removed) |  |

**All materials accessed 01/12/2018*
