# Supplementary material for: Centering the Inner Experience of Autism: Development of the Self-Assessment of Autistic Traits
Source: Autism Adulthood. 2023 Mar 13;5(1):93–105. doi: 10.1089/aut.2021.0099 (PMC10024271; doi:10.1089/aut.2021.0099)
Supplement: Supplemental data [file Suppl_MaterialS2.docx]

**2.** **Autistic Self Advocacy Network “About Autism” (2018) online publication used as a preliminary tool organize descriptors**

1. **Different sensory experiences.** For example, heightened sensitivity to light, difficulty interpreting internal physical sensations, hearing loud sounds as soft and soft sounds as loud, or synesthesia.
2. **Non-standard ways of learning and approaching problem solving.** For example, learning “difficult” tasks (e.g. calculus) before “simple” tasks (e.g. addition), difficulty with “executive functions,” or being simultaneously gifted at tasks requiring fluid intelligence and intellectually disabled at tasks requiring verbal skills.
3. **Deeply focused thinking and passionate interests in specific subjects.** “Narrow but deep,” these “special interests” could be anything from mathematics to ballet, from doorknobs to physics, and from politics to bits of shiny paper.
4. **Atypical, sometimes repetitive, movement.** This includes “stereotyped” and “self-stimulatory” behavior such as rocking or flapping, and also the difficulties with motor skills and motor planning associated with apraxia or dyspraxia.
5. **Need for consistency, routine, and order.** For example, holidays may be experienced more with anxiety than pleasure, as they mean time off from school and the disruption of the usual order of things. People on the autistic spectrum may take great pleasure in organizing and arranging items.
6. **Difficulties in understanding and expressing language as used in typical communication, both verbal and non-verbal.** This may manifest similarly to semantic-pragmatic language disorder. It’s often because a young child does not seem to be developing language that a parent first seeks to have a child evaluated. As adults, people with an autism spectrum diagnosis often continue to struggle to use language to explain their emotions and internal state, and to articulate concepts (which is not to say they do not experience and understand these).
7. **Difficulties in understanding and expressing typical social interaction.** For example, preferring parallel interaction, having delayed responses to social stimulus, or behaving in an “inappropriate” manner to the norms of a given social context (for example, not saying “hi” immediately after another person says “hi”).
