# Supplementary material for: Centering the Inner Experience of Autism: Development of the Self-Assessment of Autistic Traits
Source: Autism Adulthood. 2023 Mar 13;5(1):93–105. doi: 10.1089/aut.2021.0099 (PMC10024271; doi:10.1089/aut.2021.0099)
Supplement: Supplemental data [file Suppl_Data.docx]

**3.**  **Preliminary Item Pool of the Self Assessment of Autistic Traits for Pilot Testing at Final Stage of Development Process**

1. I process things much better one sense at a time:

An example might be: I might need to close my eyes so that I can hear.

1. It's hard for me to notice when my body needs something:

Some examples might be: I might not know that I need to go to the bathroom until it's almost too late. Or, I might not realize that I'm hurt or sick.

1. My senses are extreme. They can be intense and hard to ignore. Or, they can be weak and hard to recognize.
2. I get overwhelmed by things my body senses:

An example might be: Common sounds or smells can be so strong that it's hard to think about

anything else.

1. Doing small tasks with my hands is easy:

Some examples might be: Writing, buttoning a shirt, or tying my shoes.

1. Doing big movements with my body is easy:

Some examples might be: Riding a bike, kicking a ball, or dancing.

1. Learning new ways to move my body or my hands is easy:

An example might be: Learning a new craft or sport.

1. I like to do certain things over and over again:

Some examples might be: Flapping my hands, listening to the same song on repeat, or lining

things up a certain way.

1. I have strong urges to make certain movements, even if I don't want to:

Some examples might be: Hitting my head, or touching lamp posts.

1. I have intense, passionate interests. I like to spend as much time thinking about them as possible.
2. I notice details that other people don't.
3. I prefer to follow a routine.
4. I do my best when I know what to expect.
5. I can't always understand what people say when they talk to me. It's like my brain doesn't hear their words right away:

Some examples might be: I might need to hear the words several times.

Or, I might need to see them written down before I can understand them.

1. Using language is automatic for me:

An example might be: It is easy for me to turn what I think into words.

1. I practice what I want to tell someone in advance:

Some examples might be: Before I hang out with a friend, I type different ideas to share.

Or, before I buy ice cream, I prepare what I want to tell the cashier.

1. It is hard for me to come up with my own words. It is easier to repeat things I've

heard other people say:

An example might be: When I'm talking to someone, I might borrow responses I've heard in other

conversations.

1. It is easier for me to have conversations online or via text.
2. I make up my own ways for figuring out what people mean by their tone of voice, body language, and face:

An example might be: Instead of looking at someone's face, I look at how tense their body is.

1. It is easy for me to know what people mean by their tone of voice, body language, and face.
2. I can count on other people to understand my tone of voice, body language, and face:

Some examples might be: People know I'm mad by the expression on my face.

Or, people tell me I look happy when I feel happy.

1. In a conversation, it is hard to understand other people and communicate what I mean at the same time.
2. It is easy for me to sync socially with groups of people:

An example might be: When I am with a group of people, I automatically match their volume and

body language.

1. It is easy for me to know what other people think is important in a conversation.
2. I use strategies to figure out social situations:

An example might be: I use things like logic, lists of rules, or flowcharts.

1. I don't know how to have the friendships I want:

Some examples might be: I want friends but I don't know how to get them.

Or, I have friends but I don't know how to keep them.

Or, I have friends but I don't know how to have the kind of interactions I want.

1. I don't know how I feel until I'm overwhelmed or time has passed.
2. When I'm thinking a lot about something, it's hard for me to switch to a new topic.
3. Transitions are hard.
4. It's easy for me to break down a task or a goal into several steps.
5. I can get so focused on something that I stop being aware of the rest of the world.
6. I get overwhelmed by things that are easy for other people.
7. When I feel overwhelmed, I might have a meltdown. Or, I might need to leave.
8. When I feel overwhelmed, I might shut down:

An example might be: I might stop being able to communicate, think, or move. It might be hard to

figure out what to do.

1. I need to rest and recover after I do something really overwhelming:

Some examples might be: If I go to a party, I need to spend less time with people for the rest of

the week.

Or, if I go shopping, I need to take the rest of the day off.

1. There are times I suddenly lose skills that I used to know very well.
2. Some of my abilities vary from day to day:

Some examples might be: Some days it's easier for me to use words than on other days.

Or, some days I can tie my shoes, and other days I can't.

1. In order to show what I am capable of, I need to do things a certain way:

Some examples might be: I need extra time when I take a test.

Or, I need to do chores in a certain order.

1. I can spend hours trying to start doing something that I want to do, but no matter how hard I try, I can't get started:

An example might be: I'm hungry, but I never get around to making lunch.

1. I can't always do what I need to do, because it feels like my mind and my body are disconnected:

An example might be: I need to get lunch, but I can't make myself stand up.

1. I can't do what I need to do on my own. I need another person to help me:

An example might be: Even when I have supports like an alarm, list, or picture, I still need

someone to prompt me to go to bed.

1. I am good at recognizing patterns.
2. Daily living tasks are harder for me than reasoning or logic tasks:

Some examples might be: It is easier to program a robot than to take care of myself.

Or, it is easier for me to write a paper than to remember to turn it in.

1. Just because I can do one thing doesn't mean I can do things that other people think are similar:

Some examples might be: I might have a big vocabulary but still have trouble following

conversations.

Or, I understand advanced math. But, I make basic arithmetic mistakes.

1. I know how to take care of myself, but I forget to do things:

Some examples might be: I can forget to eat, do hygiene tasks, or take medicine.

1. It is hard for me to do all the things I need to do in the day to take care of myself. It is so hard that I wish I had additional support:

Some examples might be: Staying on top of laundry, cooking, cleaning, scheduling things, etc.

1. When something interests me, I can focus on it so much that I become an expert.
2. I am treated unfairly by others:

Some examples might be: I am bullied at school.

Or, I am mistreated at work.

1. People tell me my experiences aren't real:

An example might be: They tell me that I'm not feeling what I'm feeling.

1. People get mad at me when I don't act the way they expect me to.
2. I am always tense, or on-guard, in case social situations go wrong.
3. When other people suffer, I feel it strongly.
4. I care a lot about people being treated fairly.
5. I am direct when I communicate with people:

An example might be: I tell people exactly what I think.

1. I am a literal thinker:

An example might be: I need people to explain jokes or figures of speech to me.

1. I love some sensations so much that I can get lost in them.
2. I have a hard time getting other people's attention when I'm in a group. It is like other people don't notice me.
3. I say things I don't mean because I get stuck saying the wrong words.
